# Assessment of Night Vision Problems in Patients with Congenital Stationary Night Blindness

**DOI:** 10.1371/journal.pone.0062927

**Published:** 2013-05-03

**Authors:** Mieke M. C. Bijveld, Maria M. van Genderen, Frank P. Hoeben, Amir A. Katzin, Ruth M. A. van Nispen, Frans C. C. Riemslag, Astrid M. L. Kappers

**Affiliations:** 1 Bartiméus Institute for the Visually Impaired, Zeist, The Netherlands; 2 MOVE Research Institute, Faculty of Human Movement Sciences, VU University, Amsterdam, The Netherlands; 3 Department of Ophthalmology, VU University Medical Center, Amsterdam, The Netherlands; 4 EMGO+ Institute for Health and Care Research, VU University Medical Center, Amsterdam, The Netherlands; 5 The Rotterdam Eye Hospital, Rotterdam, The Netherlands; University Zürich, Switzerland

## Abstract

Congenital Stationary Night Blindness (CSNB) is a retinal disorder caused by a signal transmission defect between photoreceptors and bipolar cells. CSNB can be subdivided in CSNB2 (rod signal transmission reduced) and CSNB1 (rod signal transmission absent). The present study is the first in which night vision problems are assessed in CSNB patients in a systematic way, with the purpose of improving rehabilitation for these patients. We assessed the night vision problems of 13 CSNB2 patients and 9 CSNB1 patients by means of a questionnaire on low luminance situations. We furthermore investigated their dark adapted visual functions by the Goldmann Weekers dark adaptation curve, a dark adapted static visual field, and a two-dimensional version of the “Light Lab”. In the latter test, a digital image of a living room with objects was projected on a screen. While increasing the luminance of the image, we asked the patients to report on detection and recognition of objects. The questionnaire showed that the CSNB2 patients hardly experienced any night vision problems, while all CSNB1 patients experienced some problems although they generally did not describe them as severe. The three scotopic tests showed minimally to moderately decreased dark adapted visual functions in the CSNB2 patients, with differences between patients. In contrast, the dark adapted visual functions of the CSNB1 patients were more severely affected, but showed almost no differences between patients. The results from the “2D Light Lab” showed that all CSNB1 patients were blind at low intensities (equal to starlight), but quickly regained vision at higher intensities (full moonlight). Just above their dark adapted thresholds both CSNB1 and CSNB2 patients had normal visual fields. From the results we conclude that night vision problems in CSNB, in contrast to what the name suggests, are not conspicuous and generally not disabling.

## Introduction

The Schubert-Bornschein type of Congenital Stationary Night Blindness (CSNB) comprises a genetically heterogeneous group of stationary retinal disorders, caused by defective signal transmission between photoreceptors and bipolar cells. Because of this defect, the standard flash electroretinogram (ERG) is electronegative (normal a-wave, absent b-wave) [1]. Symptoms associated with CSNB are high refractive error, decreased visual acuity, nystagmus, and abnormal dark adaptation.

CSNB can be subdivided into “complete” CSNB (CSNB1) and “incomplete” CSNB (CSNB2) based on differences in ERG [2]. CSNB2 is caused by defective proteins on the synaptic terminal of photoreceptors, which are involved in continuous calcium-dependent neurotransmitter release. Therefore, the transmission of both rod and cone signals is affected but reduced activity remains present [3]. The ERG in CSNB2 shows reduced but recordable rod function and reduced cone function. CSNB1 is caused by abnormal proteins on the ON bipolar cell. Because rod signals primarily travel through ON bipolar cells, defective ON bipolar cell function leads to completely absent rod pathway signalling [4] and the ERG of CSNB1 patients show no residual rod function. CSNB segregates in X-linked (xl) and autosomal-recessive (ar) form. To date, four genes are associated with CSNB1: *NYX* (xl) [5], [6], *GRM6* (ar) [7], [8], *TRPM1* (ar) [9]–[11], and *GPR179* (ar) [12]. Two disease genes have been implicated in CSNB2: *CACNA1F* (xl) [13], and *CABP4* (ar) [14], [15].

The dark adaptation (DA) curve is a diagnostic tool used as a psychophysical measurement of night blindness [16]. The DA curve records the adaptation of rods and cones to darkness after a period of bright light stimulation (usually 5 to 10 min). Rod signal transmission is impaired in both CSNB2 and CSNB1, resulting in an abnormal DA curve with an elevated dark adapted threshold. However, the relationship between the DA curve and night vision problems is unclear. The DA curve provides no information on the visual field, while a sufficient visual field is essential for mobility and orientation. Also, the DA curve does not predict the visual functioning at light levels above the patient’s threshold.

Although the condition is named “night blindness”, not all CSNB patients experience night vision problems. We recently performed a study on 101 CSNB patients [17]. In this study, all CSNB1 patients reported night vision problems, in contrast to only 54% (31/57) of the CSNB2 patients. Other studies also reported symptomatic night blindness in all CSNB1 patients [6], [18], [19], but symptoms in CSNB2 varied: night blindness was reported in all [18], [19], in none [20]–[22] or some CSNB2 patients [13], [23]–[25]. However, the frequency and severity of night vision problems in CSNB patients have never been investigated thoroughly.

The purpose of this study was to improve rehabilitation of CSNB patients. Therefore, we assessed night vision problems in a group of CSNB patients with a questionnaire, to evaluate how often patients experience night vision problems in various situations. Furthermore, we assessed the visual functions of CSNB patients at low light intensities by means of the conventional DA curve and two other tests: the “scotopic visual field” and the “2D Light Lab”. The scotopic visual field consisted of measurements of the dark adapted threshold at different locations in the visual field. In the 2D Light Lab, we projected an image of a living room on a screen. While slowly increasing the light intensity of the projection, we asked the patients to report on the detection and recognition of the objects at different light levels.

## Materials and Methods

### Ethics Statement

The research followed the tenets of the Declaration of Helsinki. All participants were minimally 12 years of age, and we obtained written informed consent from each of the participants, and from the parents of patients under 18 years of age. Local ethical approval of the Bartiméus Institute was obtained for this procedure and this study.

### Subjects

Twenty healthy subjects (12 female, 8 male) participated as control subjects for the scotopic visual field and the 2D Light Lab. Their age ranged from 12 to 53 years, with an average of 29 years. All had normal vision and a refractive error between −5.0 D and +5.0 D, except for one subject who was highly myopic (−11D). His results were comparable to the other normal subjects.

We recruited participants from our previous study on 101 CSNB patients [17]. In this study, the phenotype data included full ophthalmic examinations and ERG. The patients were diagnosed with CSNB1 or CSNB2 based on standard ERG measurements. Subsequently, in 93% of the patients a causative mutation was found that confirmed the electrophysiologically established diagnosis; 7% of the patients had an unknown genetic defect. This study showed that the diagnostic differentiation of CSNB1 and CSNB2 can reliably be made on the basis of ERG. From this cohort, we selected patients on the basis of age and travel distance from the Bartiméus Institute. Twenty-three of twenty-six invitations were accepted.

Fourteen CSNB2 patients participated in this study. One CSNB2 patient showed unusual behaviour compared to the other patients as he walked with a cane and his mesopic visual field showed abnormalities. (The mesopic visual field is a standard test that is performed at intensities were both cones and rods are active.) Because we doubted whether his impaired visual functions could be attributed to CSNB alone, and because the patient refused further investigations, we had to remove his results from the study. The other thirteen CSNB2 patients had a normal or near normal mesopic visual field. Their average age was 24 years, their average visual acuity was 0.44 log Mar, and their average refractive error was −5.5D spherical equivalent. Individual data is given in Table 1. Subjects 2.6 and 2.10, subjects 2.7 and 2.8 and subjects 2.13 and 2.9 were brothers. Nine CSNB1 patients participated in this study. All had a normal or near normal mesopic visual field. Their age was on average 22 years, their average visual acuity was 0.23 log Mar, and their average refractive error was −7.0D spherical equivalent. Subjects 1.7 and 1.8 were brothers.

**Table 1:** Characteristics of the thirteen CSNB2 and nine CSNB1 patients that participated in the study.

	type	age [y]	gender	visual acuity [log Mar]	refractive error [D]*	nystagmus	gene mutated
1.1	CSNB1	12	male	0.12	−11.1	no	*NYX*
1.2	CSNB1	19	female	0.52	−1.9	yes	*TRPM1*
1.3	CSNB1	27	male	0.35	−13.3	yes	*NYX*
1.4	CSNB1	37	male	0.52	−11.5	yes	*NYX*
1.5	CSNB1	18	male	0.05	−4.9	yes	*unknown*
1.6	CSNB1	13	male	0.30	−5.6	yes	*TRPM1*
1.7	CSNB1	23	male	0.00	−9.0	yes	*NYX*
1.8	CSNB1	27	male	0.22	−9.6	yes	*NYX*
1.9	CSNB1	23	male	0.00	−5.2	no	*NYX*
2.1	CSNB2	16	male	0.26	−3.6	yes	*CACNA1F*
2.2	CSNB2	25	male	0.15	−4.3	no	*CACNA1F*
2.3	CSNB2	27	male	0.52	−2.9	yes	*CACNA1F*
2.4	CSNB2	31	male	0.20	−7.8	no	*CACNA1F*
2.5	CSNB2	19	male	0.30	−11.0	yes	*CACNA1F*
2.6	CSNB2	26	male	0.30	−5.8	no	*CACNA1F*
2.7	CSNB2	32	male	1.00	−9.8	yes	*CACNA1F*
2.8	CSNB2	29	male	0.40	−6.0	yes	*CACNA1F*
2.9	CSNB2	14	male	0.30	0.0	no	*CACNA1F*
2.10	CSNB2	20	male	0.22	−9.9	yes	*CACNA1F*
2.11	CSNB2	21	male	1.00	−3.5	yes	*CACNA1F*
2.12	CSNB2	18	male	0.70	−8.9	yes	*CACNA1F*
2.13	CSNB2	16	male	0.40	1.2	no	*CACNA1F*

* Refractive errors are given in spherical equivalent dioptres.

All 13 CSNB2 patients showed the typical CSNB2 ERG phenotype: reduced but recordable dark-adapted rod ERG, electronegative dark-adapted rod-cone ERG, reduced light-adapted cone ERG. All 9 CSNB1 patients showed the typical CSNB1 ERG phenotype: none recordable dark-adapted rod ERG, electronegative dark-adapted rod-cone ERG, close to normal light-adapted cone ERG. In all CSNB patients the causative mutation was found, except for one CSNB1 patient (see Table 1). The 13 CSNB2 patients and the 9 CSNB1 patients showed comparable variations in visual acuity, refractive error, and DA curve as the 62 CSNB2 and the 39 CSNB1 patients which we described in the previous study. Therefore, we assume that our cohort of patients constitutes a fairly representative group of CSNB2 and CSNB1 patients.

### Questionnaire

We developed a questionnaire based on two low luminance questionnaires available from the literature. The 35-items questionnaire of Turano et al. has been validated to monitor the independent mobility of patients with retinitis pigmentosa (RP) [26] and patients with glaucoma [27]. The 32-items questionnaire of Owsley et al. [28], [29] has been validated in patients with age-related maculopathy (ARM) to recognize the first ARM symptoms. However, RP and ARM are progressive diseases that affect the visual field, while CSNB is stationary and visual fields are normal, so not all questions were relevant for our study. Therefore, we made a selection and also added questions based on our own lab experience.

The questionnaire existed of seven parts. In Part 1, we asked the patient about the lighting conditions in his direct living environment, and the frequency of outdoor activities in summer and winter. In Part 2, the patient was asked to describe three situations in which he experienced restrictions or difficulties because of his vision at night. Part 3 dealt with means of transportation during the day, in twilight, and in the dark, and the influence of night vision problems on the choice of transportation. In Part 4 (6 items), we asked the patient how often he used assistance (a cane, another person, a flash light etc.) when walking outside in the dark. In the last three parts, we asked the patient how often he experienced a certain problem or difficulty in the dark without assistance: “outdoor problems” (Part 5; 12 items), “indoor problems” (Part 6; 6 items), and “general problems” (Part 7; 6 items). Response options for Part 4–7 were: never, sometimes, regularly, often, always (scored 1 for “never” up to 5 for “always”). The patient was asked to tick “not applicable” if an activity was never performed, or only performed *with* assistance. The complete questionnaire can be found in the supplemental data (Appendix S1).

The results from the questionnaire are given as follows: we give a summarized description of the answers of the first three parts of the questionnaire. To visualize the distribution of response categories for separate items of Parts 4–7, we scored how many of the five possible categories were given per item by CSNB1 and CSNB2 patients, respectively. We give the first quartile, the median and the third quartile of the answers per question, i.e. the 25%, 50% and 75% cut off of the ranked data. We excluded patients who ticked “not applicable”, which occurred no more than twice per question.

### Dark Adaptation Curve

We recorded dark adaptation curves (DA curve) with a Goldmann-Weekers Dark Adaptometer in a completely darkened room after the subjects were light-adapted with a Ganzfeld background (about 1000 cd/m^2^) for 10 minutes. The binocular threshold was measured during 20 to 25 minutes of dark adaptation, using an 11^o^ off-central white circular target with a diameter of 56 mm, or 11°. The stimulus was presented at a 0.5 Hz flicker rate. The manufacturer of Goldmann-Weekers Dark Adaptometer supplies examples of normal data for various age ranges. The results of our patients were compared to the standard DA curve of subjects between 20 and 40 years old.

### Scotopic Visual Field

We measured binocular static scotopic visual fields using the Perimeter Octopus 900 (Haag-Streit AG, Switzerland). We adapted the perimeter to make it suitable for this purpose. The subject was positioned in front of the bowl by a head and chin rest. The central green fixation dot was filtered by a red filter so that a very dim red fixation dot remained, which was still visible to all subjects. The measurements were performed in complete darkness. Background light was turned off and black tape suppressed visible red light from infrared LEDs. We also covered the buttons and the screen in and outside the Octopus bowl and dimmed the computer screen with filters to minimize scatter light.

**Figure 1:**
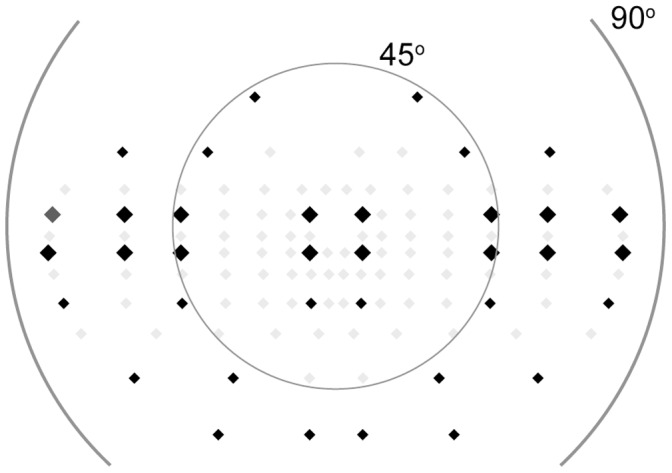
Target locations of the scotopic visual field. The scotopic visual field locations (black) were based on the locations used in the Esterman test (black and grey). The large diamonds represent the locations that were used to determine the homogeneity of the visual field by comparing the average threshold at 7°, 45°, 60°, and 75°.

We chose target locations from the Esterman visual field [30], because it is a very wide visual field test. We removed several target locations to keep measurement time acceptable (<15 min). Fig. 1 shows the standard Esterman target locations (grey) and the 36 selected locations (black). To analyse the homogeneity of the threshold across the visual field, we averaged the thresholds of four locations at 7°, 45°, 60° and 75° on the horizontal axes (large black diamonds in Fig. 1).

We used the largest size stimulus (64 mm^2^, Goldmann V) to rule out visual acuity as a determining factor. We used the dimmest stimulus (blue, λ = 440 nm +/−25 nm) and minimized the flash duration to 100 ms. The maximal intensity (0 dB) of the stimulus was 16 apostilb, i.e. 5.1 cd/m^2^. The stimulus could be dimmed 47 dB. Because the minimal intensity was still easily detectable by control subjects and CSNB2 patients, for these subjects we filtered the stimulus with an extra 3.0 log units of intensity neutral density filter. In the CNSB1 patients, we measured the scotopic visual field *without* the extra filter. In five CSNB1 patients we also completed a test *with* the filter. In these five patients, the threshold could be determined in both tests at 118 target locations. The values differed on average 28 dB, standard error 0.2 dB. Thus, the measuring range in control subjects and CSNB2 patients was 28 to 75 dB, while in the CSNB1 patients it was 0 to 47 dB. If the brightest stimulus (28 dB or 0 dB, respectively) could not be detected, the measurement of that target location was removed from the data set.

The measurement of the scotopic visual field started in the four locations at 7° at an intensity of 52 dB (with filter) or 24 dB (without filter). If the subjects did not respond to the first stimulus, the intensity was increased in a 6 dB step. Thereafter, the process continued with brighter spots in steps of 8 dB until the subject perceived the stimulus and pressed the button for a “yes”. Then, the procedure continued at all locations but in a random order, in a one-up one-down staircase method with decreasing step size. The initial stimulus intensity at these locations started at an intensity higher than that of the threshold, as determined in the four locations at 7°. The stimulus intensity subsequently decreased in 4 dB intensity steps until the subjects no longer perceived the stimulus. We repeated the procedure in 2 dB steps in the opposite direction, and finally again in 1 dB steps to determine the threshold with a nominal accuracy of +/−1 dB.

We statistically compared the thresholds found in the control subjects, the CSNB2 patients, and the CSNB1 patients. We first averaged the thresholds per subject and then compared the groups by One Way ANOVA and subsequent Bonferroni corrected Post Hoc tests for pairwise multiple comparisons.

### 2D Light Lab

**Figure 2:**
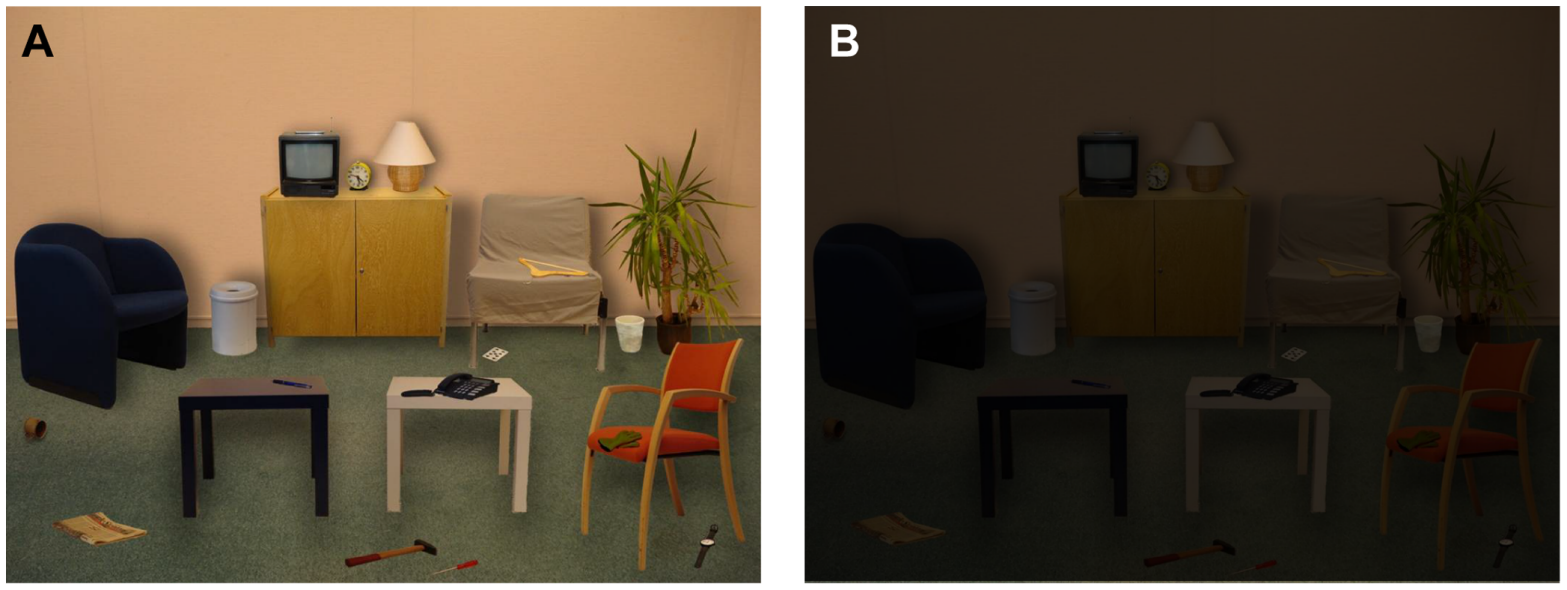
The constructed image of a living room used in the 2D Light Lab. The image contained 22 everyday objects that varied in size, colour and contrast. The objects were (from left to right and top to bottom): small television, alarm clock, table-lamp, armchair, trashcan, cupboard, bench, coat hanger, vase, plant, playing card, mug, dark table, pencil, white table, telephone, chair, pair of gloves, newspaper, hammer, screwdriver and watch. The left image (A) shows the image at full intensity, the right image (B) shows the image at a low intensity.

The two dimensional (2D) Light Lab was derived from the original three dimensional (3D) Light Lab [31] which consists of a real living room filled with daily objects. In the 3D Light Lab, patients are asked to describe the objects they detect and recognize at increasing light levels. The 2D Light Lab consisted of an image of a living room that was projected on a screen. The image used in these experiments was constructed from separate photo’s using photo-editing software (Corell Paintshop Photo Pro X3), which made it easier to control intensity and contrast. The image, shown in Fig. 2, contained 22 everyday objects that varied in size and colour. The distance between screen (4∶3, 2.40 m, Projecta) and projector (Sanyo PLC-XP100L, 3LCD, XGA) was 4 m. The subject sat on a chair 4 m from the screen, at an angle of 10° from the midline between projector and screen. Apart from the light from the screen, the room was completely dark. We used a retro reflective screen with a directed reflectivity a factor 2.4 larger than that of a standard white screen, so that the luminance in the direction of the projector was high, but indirect light scattering from the walls was minimized.

From the position of the subject, the maximum luminance measured on the screen was 2.8 log cd/m^2^. This was measured on the table-lamp which was the brightest object of the image. This maximal luminance is comparable to a white object (reflection factor 0.8) in a room with an illuminance of 2600 lux. We adjusted the luminance of the screen by placing neutral density (ND) filters in front of the projector. We combined a 3.0, 2.0 or 1.0 log units ND filter with a 0.5 log units ND filter. In addition, the subject wore goggles with 3.0 log ND filters during the first part of the experiment, and then removed the goggles. To prevent condensation of the goggles, two tubes were placed underneath the goggles and connected to a small air pump.

At the start of the experiment, the light level was maximally dimmed by 6.5 log units of intensity. Under these conditions, the luminance of the brightest object in the 2D Light Lab is comparable to the luminance of a white paper in starlight, i.e. −3.7 log cd/m^2^ [32]. We then increased the light level to maximal intensity in 14 steps of 0.5 log units. At 3.5 log units, the luminance of the brightest object is comparable to the luminance of a white paper in full moonlight, i.e. −0.7 log cd/m^2^ [32].

During the experiment, we asked the subject to describe his observations. We noted detection (d, see ‘something’, usually roughly the outline of the object) and recognition of the 22 objects per intensity step. For each step, the subject had several minutes to adapt to the light level and to inspect the image thoroughly.

For each intensity, the cumulative number of objects that was detected or recognized was divided by the total number of objects. This data plotted against the intensity resembled a logistic psychometric function: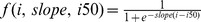. Here, *i* is the intensity, *slope* is the slope of the curve, and *i50* is the intensity at which 50% of the objects were detected or recognized. We assumed that the subjects could detect and recognize all objects at maximal intensity. We fitted a psychometric function to the light lab data involving detection (d) and recognition (r) of each subject. We determined and statistically compared slope_d_, slope_r_, i50_d_, and i50_r_ between the control subjects, the CSNB2 patients, and the CSNB1 patients, using a One Way ANOVA test and subsequent Bonferroni corrected Post Hoc tests for pairwise multiple comparisons. Finally, we performed simple linear regression analyses between i50_d_, i50_r_, and visual acuity and determined the Pearson correlation coefficients.

### Testing Procedure

For the control subjects, the complete experiment consisted of the scotopic visual field test and the 2D Light Lab test. Before the start of the scotopic visual field test, the subject dark adapted for 20 minutes by wearing occluding goggles. Afterwards, the subject was asked to again put on the occluding goggles. The examiner then guided the blindfolded subject to the room with the 2D Light Lab test. Before the start of this test, the subject replaced the occluding goggles by the 3.0 log goggles, while keeping his eyes closed. The examiner assisted under minimal light levels.

The procedure for the patients was more extensive. We asked the patients to fill in the questionnaire at home. Before starting the experiments, the examiner shortly discussed the questionnaire with the patients, to make sure they had answered and correctly understood all questions. The experiment started with the dark adaptation curve, which was recorded for 20 to 25 min. Subsequently, the patient was asked to put on the occluding goggles. The examiner then guided the blindfolded patient to the room where the scotopic visual field test was performed. Because the patient was already dark adapted, the experiment could start immediately. Thereafter, the tests were performed in the same manner as in the control subjects.

## Results

### Questionnaire

In Part 1, both CSNB2 and CSNB1 patients reported going out almost as often or equally often in winter as in summer time. In Part 2, we asked the patients to describe three situations in which they felt restricted or bothered because of their vision at night. Several CSNB2 patients described mobility problems, for instance having to walk or cycle without enough streetlight. CSNB1 patients in addition described problems recognizing persons or finding their seat during social events like going out to the pub, cinema, or at a campsite.

In Part 3, all CSNB2 and CSNB1 patients reported going out on foot during the day, at twilight, and at night (if necessary with a flashlight), except for one young CSNB1 patient who was not allowed to go out alone at night because of his age. He and one other CSNB1 patient only cycled during the day, and two CSNB2 patients did not cycle at all (the bicycle is the most frequently used means of transportation in the Netherlands). All other patients cycled under all circumstances. Five CSNB2 and three CSNB1 patients had a car driving licence but two of the CSNB2 patients were only permitted to drive during the day. Only one CSNB1 patient chose to not obtain his driving license because of his vision at night, although he met the criteria for a driving license during the day. The other patients had other reasons for not having a driving license: they were either too young, did not meet the minimal visual acuity criterion, or preferred other means of transportation. One CSNB2 patient reported to be a truck driver.

**Figure 3:**
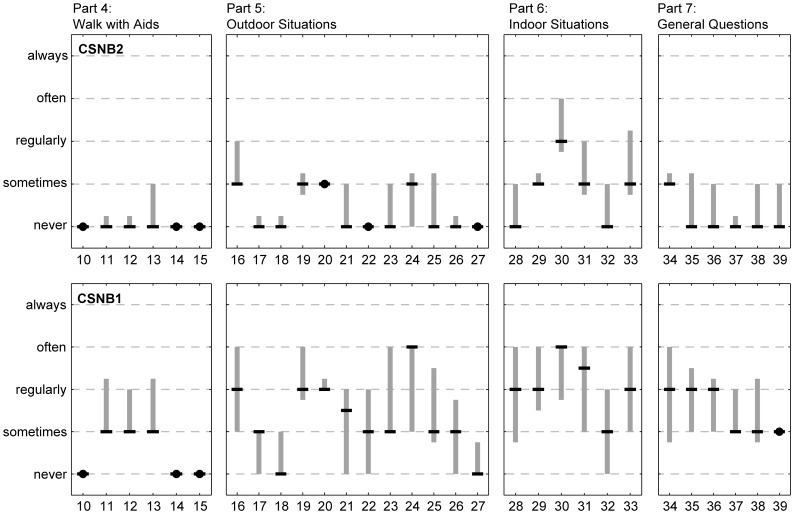
Results of Parts 4 to 7 of the questionnaire. Top: answers of CSNB2 patients. Bottom: answers of CSNB1 patients. The black horizontal lines indicate the medians. The grey vertical lines extend from the first quartile to the third quartile and thus indicate the range of the mid 50% ranked answers. A black dot is used when the mid 50% ranked date contained one answer only.

Fig. 3 shows the first quartile, the median and the third quartile of ranked response options the patients gave to Parts 4 to 7 of the questionnaire, dealing with assistance or devices, outdoor problems, indoor problems, and general problems. The results show that CSNB2 patients answered most questions about problems with “never” or “sometimes”, while CSNB1 patients experienced problems more frequently. None of the CSNB2 and CSNB1 patients used a cane when walking in the dark (Q10), but CSNB1 patients sometimes used other aids (Q11–13). One CSNB1 patient described that he sometimes used his phone as a flashlight (Q14), and one CSNB2 patient described to always seek for the best illuminated areas (Q15). Both CSNB2 and CSNB1 patients experienced less problems moving about or finding their way in a familiar environment (Q17,18) compared to an unfamiliar environment (Q19,20). None of the CSNB2 patients and only two of the CSNB1 patients reported going out less often than they wanted because of their vision at night (Q27). Both CSNB2 and CSNB1 patients reported more problems in Part 6 (Indoor problems like recognizing faces or reading a book) compared to Parts 4, 5 and 7. Finally, CSNB1 patients felt blind at night (Q36), insecure at a social event (Q37), restricted because of their vision at night (Q38) and dependent on others in dark circumstances (Q39), but at different frequencies. CSNB2 patients mostly reported “never” or “sometimes” to these questions.

### Dark Adaptation Curve

**Figure 4:**
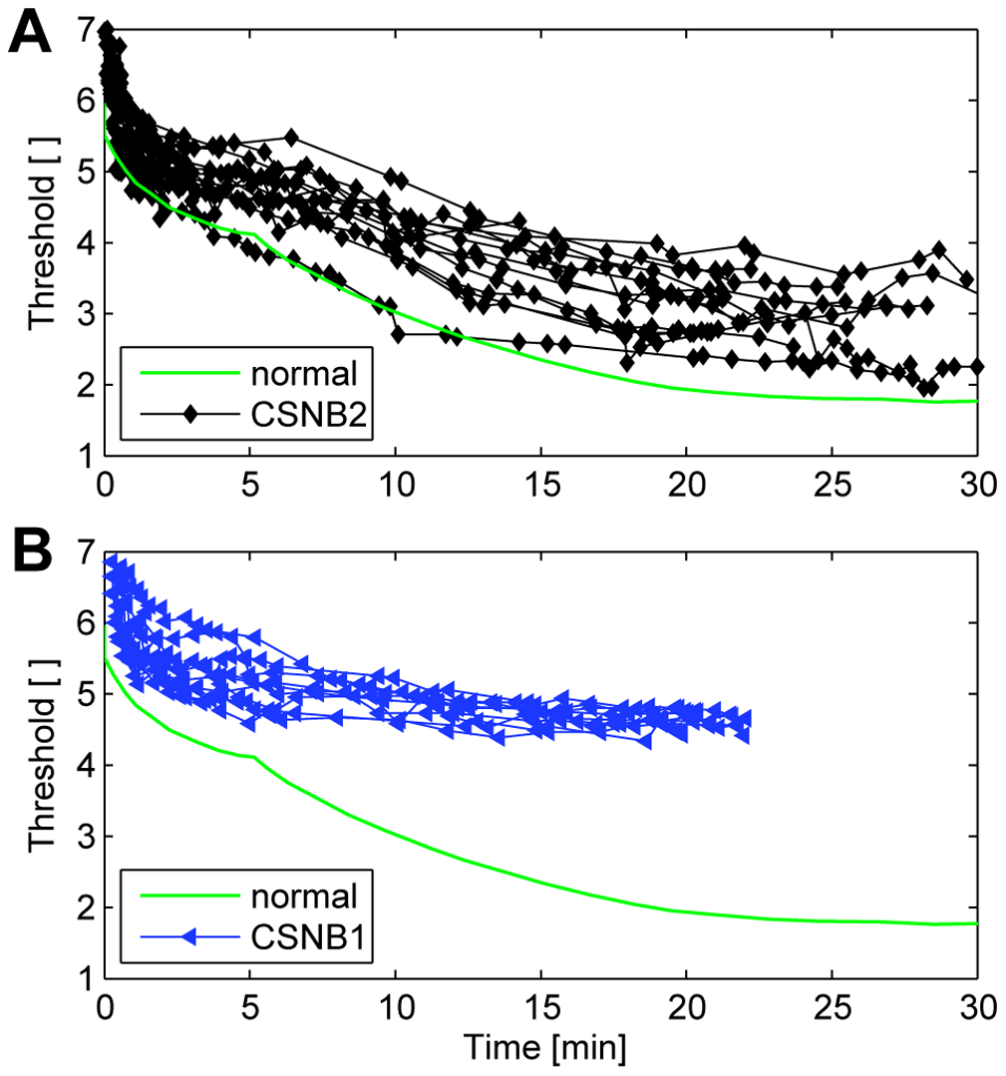
Dark adaptation curves of the CSNB2 patients (A) and CSNB1 patients (B), and a control subject (green curve). The normal DA curve shows a biphasic form, with an early cone-mediated phase and a later rod-mediated phase. The DA curves of the CSNB2 patients also showed such a biphasic form. Their final thresholds were variably elevated. The DA curves of the CSNB1 patients only showed a cone-mediated phase. Their final thresholds were all approximately 3 log units elevated.

In normal subjects, the DA curve shows a biphasic form, with an early cone-mediated phase during the first 5 min and a later rod-mediated phase (Fig. 4, green solid curve). The dark adaptation curves of the CSNB2 patients also show such a biphasic form but the final threshold was elevated, see Fig. 4. The threshold after 5 min varied between 4.0 and 5.5 log units, a range of 1.5 log units, and after 25 min between 2.2 and 3.7 log units, a range of 1.5 log units. In contrast, in the CSNB1 patients the variation in the threshold was large at the beginning of the dark adaptation curve, but decreased as the dark adaptation continued. The threshold after 5 min varied between 4.8 and 5.8 log units, a range of 1 log unit, but the final threshold after 20 min was found between 4.55 and 4.8 log units, a range of only 0.25 log units. Compared to the final threshold of 1.8 log units in a control subject, the final threshold elevation was between 0.4 and 1.9 log units in the CSNB2 patients and about 3 log units in the CSNB1 patients.

### Scotopic Visual field

**Figure 5:**
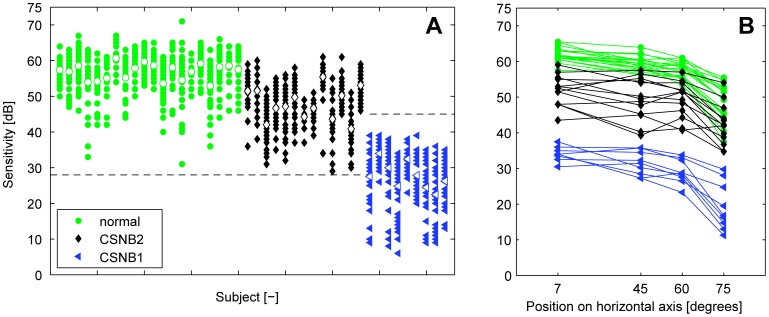
Scotopic visual field results of the normal subjects, the CSNB2 patients, and the CSNB1 patients. A: The threshold found at each location of the scotopic visual field, plotted per subject. The open markers represent the average threshold found in that subject. The dashed lines indicate the measuring range, which was 28 to 75 dB in control subjects and CSNB2 patients, and 0 to 47 dB in CSNB1 patients. We found slightly elevated thresholds in CSNB2 patients compared to the thresholds of normal subjects. The thresholds were more elevated in the CSNB1 patients. B: The averaged thresholds of four locations at 7°, 45°, 60° and 75° on the horizontal axes per subject. Thresholds were slightly elevated towards the far end of the visual field in control subjects. We found an equal decline in the control subjects, the CSNB2 and the CSNB1 patients.

In Fig. 5A we plotted the thresholds found at the 36 target locations per subject. Occasionally, the brightest stimulus could not be detected by the subjects. The measurement of that target location was removed from the data set. This happened for one target location in one control subject, in one target location in a CSNB2 patient, and in two target locations in another CSNB2 patient. The average thresholds per control subject (open green circles) were found between 53 dB and 61 dB, with an average of 57 dB. The CSNB2 patients had an average threshold per subject (open black diamonds) between 41 dB and 55 dB, with an average of 48 dB. The CSNB1 patients had an average threshold per subject (open blue triangles) between 22 dB and 34 dB, with an average of 28 dB. The One-Way ANOVA test showed significant differences between the average thresholds found in each group, *F* = 230.0, *p*<0.0001. Bonferroni corrected Post Hoc tests showed that all three groups were significantly different from one another (for all three pairs, p<0.0001).

We found a small decline of the threshold from 7° to 60° and a stronger decline between 60° and 75°, see Fig. 5B. For the control subjects, the average thresholds at 7°, 45°, 60° and 75° were 62, 59, 58 and 49 dB respectively. In the CSNB2 patients, these were 52, 50, 49 and 42 dB and in the CSNB1 34, 31, 29 and 19 dB.

### 2D Light Lab

**Figure 6:**
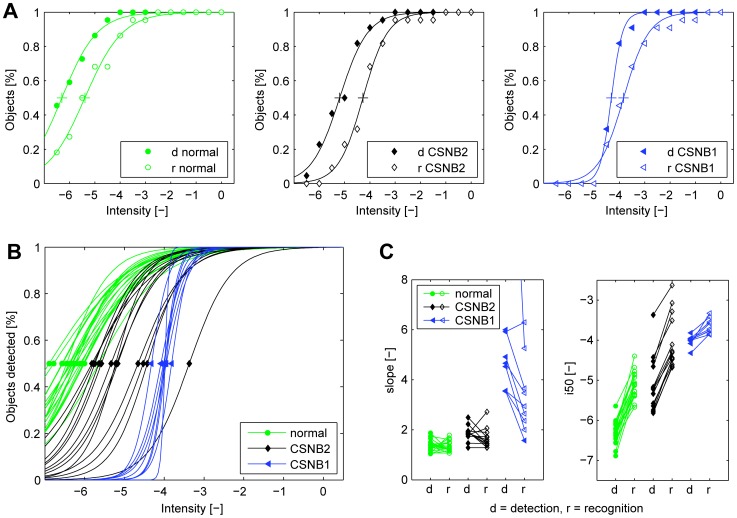
2D Light Lab results of the normal subjects, the CSNB2 patients, and the CSNB1 patients. **A**: Three representative examples of the results from the 2D Light Lab. The figure shows the cumulative of the relative number of objects detected or recognized, the fit of the psychometric curve, and the crossmarks that indicate the intensity at which 50% of the objects were detected or recognized (i50_d_ and i50_r_). **B**: The psychometric fit to the cumulative of objects detected at increasing light levels in the 2D Light Lab for each subject. The green dots (control subjects), black diamond (CSNB2 patients) and blue triangles (CSNB1 patients) in each fit indicate i50_d_ and i50_r_. **C**: The parameters (slope, left) and (i50, right) of the psychometric fit to the 2D Light Lab results of all subjects. Overall we found equal slopes for detection and recognition in control subjects and in CSNB2. However, in the CSNB2 patients the curves were shifted toward higher intensities. The two curves of the CSNB1 patients were steeper, closer together and shifted towards higher intensities compared to control subjects.

The 2D Light Lab results of a representative control subject, a CSNB2 patient, and a CSNB1 patient are given in Fig. 6A. It shows the cumulative number of objects detected (filled symbols) and recognized (open symbols), and the fit of the two psychometrical curves. The steepness of the two curves of the CSNB2 patients were almost equal to those of the control subjects, but the curves are shifted toward higher intensities. The two curves of the CSNB1 patients were also shifted towards higher intensities, and in addition steeper and closer together compared to control subjects.

Fig. 6B shows the psychometric curves for detecting objects of all subjects. As can be seen, the curves for detection of the CSNB1 patients were steeper compared to those of the control subjects and CSNB2 patients. Furthermore, the position of the curves on the horizontal axis (i50_d_) varied but little among CSNB1 patients and the most among CSNB2 patients. I50_d_ and i50_r_ varied over a range of 0.5 and 0.5 log units resp. for the CSNB1 patients, and 2.4 and 2.1 log units resp. for the CSNB2 patients.

Fig. 6C shows the values of the steepness of the psychometrical curves for detection (slope_d_) and recognition (slope_r_), and of the intensities at which 50% of the objects were detected (i50_d_) and recognized (i50_r_). We performed One-Way ANOVA tests and Bonferroni corrected Post Hoc tests to analyse the differences of these variables between control subjects, CSNB2, and CSNB1 patients, see Table 2. In the CSNB1 patients, there was one outlier (slope_d_ = 20.4) that was removed before further analyses. We found that the steepness of the curves (slope_d_ and slope_r_) of the CSNB1 patients differed significantly from those of the control subjects and CSNB2 patients. Slope_d_ was just significantly different (*p*<0.05) and slope_r_ was not significantly different between the control subjects and CSNB2 patients. The position of the curves on the horizontal axis (i50_d_ and i50_r_) were significantly different between all three groups, except for i50_r_ which was not significantly different between the CSNB2 and CSNB1 patients. Finally, we found that the curves for detection and recognition were significantly closer together in the CSNB1 patients compared to the control subjects and the CSNB2 patients (see Tabel 2, i50_r_–i50_d_).

**Table 2:** Statistical analyses of slope_d_, slope_r_, i50_d_, i50_r_, and the difference between i50_d_ and i50_r_ (i50_r_–i50_d_) between control subjects, CSNB2, and CSNB1 patients.

	slope_d_	slope_r_	i50_d_	i50_r_	i50_r_–i50_d_
overall effect	*F(2,38) = *127.7 **	*F(2,38) = *20.1**	*F(2,38) = *79.4**	*F(2,38) = *39.8**	*F(2,38) = *26.9**
control subjects - CSNB2	*	n.s.	**	**	n.s.
control subjects - CSNB1	**	**	**	**	**
CSNB2 - CSNB1	**	**	**	n.s.	**

Analyses were performed through One-Way ANOVA tests (overall effect) and Bonferroni corrected Post Hoc tests for pairwise multiple comparisons.

** *p*-value <0.001.

* *p*-value <0.05.

n.s. not significantly different.

The objects that were first detected and recognized were the larger objects and the objects with a high contrast with the environment: the table-lamp, the armchair, the cupboard, and the dark and white table. But in fact, the screen itself was the first object to be detected. Remarkably, although not systematically recorded, the CSNB1 patients did not see the screen at all and therefore did not know where to look until just before the first objects were detected. The objects that were last detected and recognized were small and had a low contrast with the environment: the coat hanger, the pencil, the pair of gloves, the mug, and the screwdriver.

**Figure 7:**
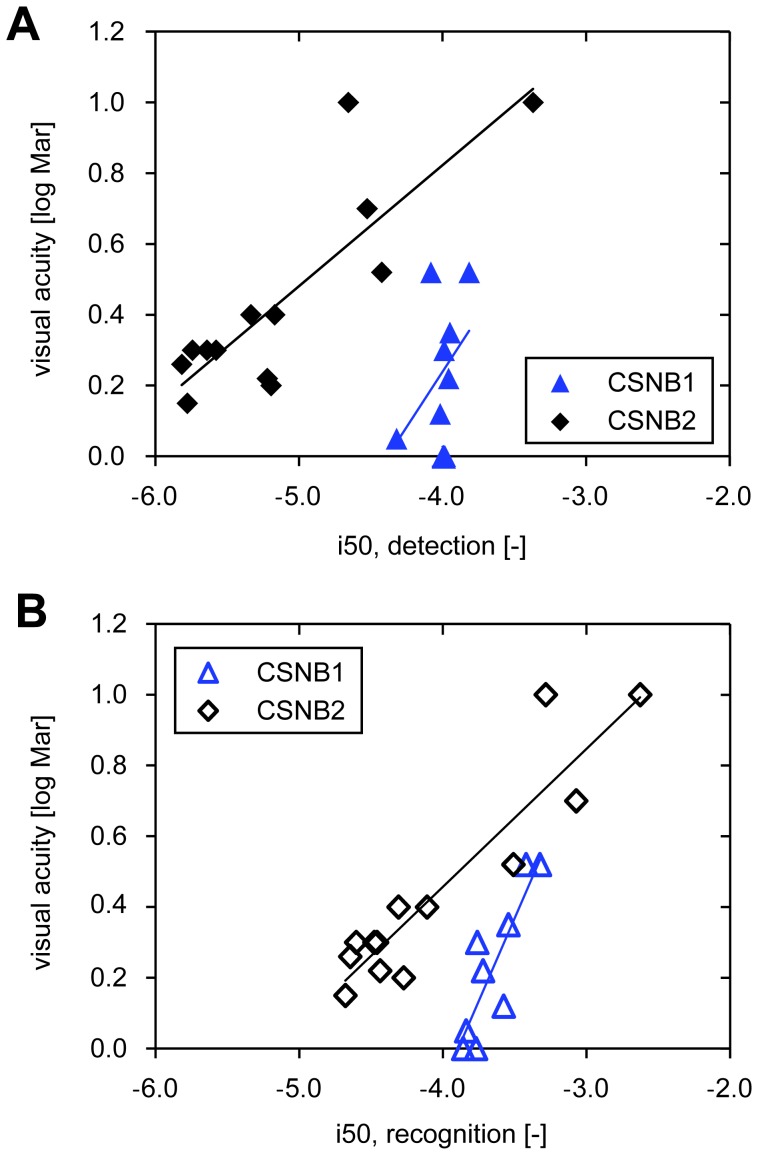
Linear regression between the intensity at which 50% of the objects were detected (i50_d_) or recognized (i50_r_) and the visual acuity. The lines resemble the simple linear regression fits. A: The Pearson correlation coefficient for i50_d_ and the visual acuity was *R* = 0.84, p<0.01 for the CSNB2 patients, and *R* = 0.41, p>0.05 for the CSNB1 patients. B: The Pearson correlation coefficient for i50_r_ and the visual acuity was *R* = 0.92, p<0.01 for the CSNB2 patients, and *R* = 0.86, p<0.01 for the CSNB1 patients.

Fig. 7A shows the linear regression between the intensity at which 50% of the objects were detected (i50_d_) and the visual acuity. The Pearson correlation coefficient was significant for the CSNB2 patients: *R* = 0.84, *p*<0.01, but not for the CSNB1 patients: *R* = 0.41, *p*>0.05. In contrast, we found a significant Pearson correlation coefficient for both patient groups between the intensity at which 50% of the objects were recognized (i50_r_) and the visual acuity (Fig. 7B). For the CSNB2 patients we found: *R* = 0.92, *p*<0.01, and for the CSNB1 patients: *R* = 0.86, *p*<0.01. The four CSNB2 patients with the poorest visual acuity needed the most light to detect and recognize the objects. In contrast, i50_d_ and i50_r_ found for the CSNB1 patients differed little, while the patients had variable visual acuities.

## Discussion

In this study we assessed night vision problems in 13 CSNB2 patients and 9 CSNB1 patients through a questionnaire, and three scotopic tests: a DA curve, a scotopic visual field and a 2D Light Lab. We found several differences between the CSNB2 and the CSNB1 groups.

The CSNB2 patients answered most questions from the questionnaire regarding problems with “never” or “sometimes”. They did not recognize the situations described in the questionnaires as problematic and did not feel blind at night. They more frequently reported to have difficulties reading a paper or a book in an insufficiently lighted environment (Q21). Therefore, in CSNB2 patients poor visual acuity appears to be more disabling than night blindness.

The scotopic test results of the CSNB2 patients showed similarities to those of the control subjects, although there were variations among patients. The dark adaptation curves of the CSNB2 patients showed a biphasic form, similar to the DA curve of normal subjects. However, their final thresholds were 0.4 to 1.9 log units elevated. Their scotopic visual field showed a slightly decreasing threshold from mid to end, just as in control subjects, but with an elevated absolute threshold. The 2D Light Lab results of CSNB2 patients and control subjects showed a comparable number of intensity steps to detect and recognize all objects (small difference in slope_d_ and slope_r_). However, the intensity at which 50% of the objects were detected and recognized (i50_d_ and i50_r_) were higher in patients and varied over a range of over 2 log units. The minimal night vision problems reported by all CSNB2 patients may therefore be explained by their relatively intact visual fields, and the similarity of the DA curves and 2D Light Lab results compared to control subjects. The increase in thresholds means that only in very dark conditions (i.e. starlight) difficulties may be expected.

We also compared the results of three pairs of CSNB2 brothers. Their differences in scotopic tests results were comparable to those of unrelated CSNB2 patients. The brothers also mentioned differences in their experience of visual problems in the dark. This indicates that other genetic, environmental factors [13], [21] or personal factors may be more important in determining the severity of night vision problems in CSNB2 than different mutations in the causative disease gene.

The results of the questionnaire showed that CSNB1 patients varied in their experience of difficult situations. They also answered variably on the questions how often they felt blind at night (Q27) and restricted because of their vision at night (Q29). The variation among CSNB1 patients may partially be explained by variation in visual acuity. Overall, all CSNB1 patients experienced night vision problems, but we would not describe them as severe.

Despite the variable answers on the questionnaire, the scotopic test results in CSNB1 patients were very similar. The first part of the DA curve differed between subjects, but the final elevated thresholds lay close together, between 2.85 and 3.1 log units. This has been described before, without an explanation [2]. Also, their scotopic visual fields were very similar as were the results from the 2D light lab. Because the CSNB1 patients had different mutations, genotype appears not to be related to scotopic functioning.

The scotopic field of the CSNB1 patients showed a minimal decline of the thresholds from the middle towards 75°, comparable to the decline found in control subjects. This means that the visual fields in CSNB1 patients were relatively intact, albeit with increased thresholds. In the 2D Light Lab, CSNB1 patients were completely blind at the lowest light levels (starlight). However, above a certain light level, they went from seeing nothing to seeing almost everything in one or two 0.5 log intensity steps. The light level at which the CSNB1 patients recognized and detected half of the objects corresponds to full moonlight illuminance. The light level at which all objects were recognized and detected approached that of the control subjects. Our results from the scotopic visual fields and the 2D light lab may explain the limited night vision problems in CSNB1. CSNB1 patients are blind by starlight, but when the light level exceeds a certain minimum, their visual field is normal and they can detect and recognize all objects equally well as persons with normal vision. In the western world, most villages, cities, streets and highways are very well illuminated and so the light level is high enough for CSNB1 patients.

In CSNB2, the transmission of both rod and cone signals is affected but reduced activity remains present [3]. Our study shows that the remaining scotopic functions vary between CSNB2 patients, suggesting that the extent to which signal transmission in CSNB2 is affected varies. This may explain the correlation between the visual acuity and the intensity at which 50% of the objects were detected (i50_d_) or recognized (i50_d_). Possibly, more severely affected signal transmission results in poorer visual acuity (cone system), *and* more light needed to see objects (rod system). In CSNB1, defective ON bipolar cell function leads to absent rod pathway signalling [4]. In our study, we found impaired but equal scotopic functions in CSNB1 patients: DA thresholds and scotopic visual fields all showed highly elevated thresholds. A previous study on dark adapted perimetry concluded that the scotopic visual field of a CSNB patient (probably CSNB1) was determined by the cone system only [33]. Our results confirm that rod signal transmission is completely blocked in CSNB1, with no variations in severity among patients.

In the 2D light lab, both control subjects and CSNB2 patients detected and recognized objects at very low intensities. This suggests that the rod system determines their detection and recognition curves. In contrast, the cone system most likely determines the detection and recognition curves of the CSNB1 patients, as they detected and recognized objects at higher intensities. However, in the CSNB1 patients we not only found higher values for i50_d_ and i50_r_, but also for slope_d_ and slope_r_, compared to the control subjects and CSNB2 patients. The question rises why the rod system induces a relatively low slope_d_ and slope_r_ compared to the cone system. Our hypothesis is that the rod system is very sensitive at low intensities, but at the expense of detail, while at higher intensities the rod system desensitizes but improves in signal to noise ratio. This hypothesis explains our observation that subjects only saw large or high contrast objects at low intensities. Furthermore, it may also explain the 1 log unit intensity shift between the detection curve and recognition curve determined by the rod system: more details are needed to recognize the object after it has been detected. In contrast, the cone system operates only at a certain minimal intensity but always at a high signal to noise ratio. Therefore, above the cone system threshold all objects can be detected and recognized.

The DA curve is considered the defining psychophysical measurement of night blindness [16]. More severe night vision problems are expected with more elevated thresholds. However, in our study CSNB1 patients had highly elevated DA thresholds (3 log units), but did not experience severe night vision problems, as assessed by the questionnaire. This may be explained by their relatively intact wide scotopic visual fields and the results of the 2D Light Lab, showing that they immediately regained vision at intensities just above the threshold. Thus, the combination of the three scotopic tests explained the results from the questionnaire. The scotopic visual field test and the 2D light lab test may be of value in predicting night vision problems in patients. Furthermore, the 2D light lab, as did the original 3D light lab, may be very useful in demonstrating to both patients and relatives the consequences of their disorder [31], [34]. However, further studies on scotopic tests in other retinal disorders are needed to confirm their clinical use in objectively assessing night blindness.

With this study, where we used two scotopic tests and a questionnaire in addition to the conventional DA curve, we add to the evidence that CSNB2 patients experience none or hardly any symptoms of night blindness. Furthermore, we showed that CSNB1 patients do experience night vision problems, but only in very dark circumstances (below full moonlight illuminance). Therefore, in the modern western world, CSNB1 patients need little adaptations in daily life routine. Rehabilitation centres and parents of young patients need to know that CSNB2 patients have no extra difficulty at night compared to day time, and that CSNB1 patients can move around in the dark independently as long as there is enough street lighting. Ophthalmologists need to know, that because of almost absent symptoms of night blindness in CSNB2 and mild symptoms in CSNB1, the name congenital stationary *night blindness* may lead to misunderstanding when explaining the condition to parents and patients. As previously stressed by Riemslag [34], names of disorders should not refer to (possible) reduced functions. We would recommend to change the name CSNB into more neutral terms related to the true retinal deficiency, for instance “ON-bipolar deficiency” for CSNB1 and “photoreceptor synapse deficiency” for CSNB2.

## Supporting Information

Appendix S1 **The complete questionnaire (Parts 1 to 7).**
(DOC)
