# Supplementary material for: Assessment of Night Vision Problems in Patients with Congenital Stationary Night Blindness
Source: PLoS One. 2013 May 3;8(5):e62927. doi: 10.1371/journal.pone.0062927 (PMC3643903; doi:10.1371/journal.pone.0062927)
Supplement: Appendix S1 — The complete questionnaire (Parts 1 to 7). (DOC) [file pone.0062927.s001.doc]

Part 1

1. Not every living environment is well lightened. For example, in the country side there are often rarely or no streetlights. In the city there is often so much lighting that it is never completely dark. How would you describe the lighting in your direct living environment?

 lighting is more than sufficient

 lighting is sufficient

 lighting is moderate

 lighting is rare

 there is no lighting

1. In wintertime, the sun sets early in the evening. In winter, how often do you go out alone per week after work or school?

 less than once per week

 about twice per week

 about three times per week

 about four times per week

 more than four times per week

1. In summertime, the sun sets late in the evening. In summer, how often do you go out alone per week after work or school?

 less than once per week

 about twice per week

 about three times per week

 about four times per week

 more than four times per week

Part 2

1. Describe three situations in which you feel restricted or that require effort in your activities because of your vision at night.

1.

2.

3.

Part 3

In the next table, several ways of traffic participation are given. Please tick the ways in which you participate in traffic, and under which lighting conditions. You can tick multiple options. In addition, please tick whether your choices are affected by your vision at night.

|  |  | By foot | bike | Moped / motorcycle / scooter | Scoot-mobile / disabled vehicle | Car | Other namely: |
| --- | --- | --- | --- | --- | --- | --- | --- |
|  |  | 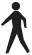 | 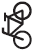 | 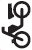 |  | 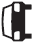 |  |
|  | Daytime |  |  |  |  |  |  |
|  | In twilight or more than sufficient streetlight |  |  |  |  |  |  |
|  | In the dark (but with a flashlight/ bicycle light/ headlight) |  |  |  |  |  |  |
|  | I do ***not*** use this means of transportation in twilight or in the dark  ***Because of your vision at night*** |  |  |  |  |  |  |
|  | I do not use this means of transportation because of other reasons |  |  |  |  |  |  |

Part 4

In the next table, several strategies are given that you may use when you walk outside in the dark. Please read every strategy and tick how often you use this strategy. N.a. represents “not applicable”.

|  | When I walk/want to walk outside in the dark… | never | sometimes | regularly | often | always | n.a. |
| --- | --- | --- | --- | --- | --- | --- | --- |
|  | …I use a cane |  |  |  |  |  |  |
|  | …I make sure someone goes with me |  |  |  |  |  |  |
|  | …I go from lamppost to lamppost |  |  |  |  |  |  |
|  | …I take a flashlight with me |  |  |  |  |  |  |
|  | …I use a different kind of aid, namely: |  |  |  |  |  |  |
|  | …I take a different kind of action, namely: |  |  |  |  |  |  |

Part 5

In the next table, several situations are described that could cause problems. Please read every situation and tick how often this situation leads to problems for you. Assume that you do ***not use an aid*** (cane, assistance, guide dog). Use n.a. if you only perform an activity *with* assistance.

The next items consider ***outdoor*** situations

|  | When it is dark... | never | sometimes | regularly | often | always | n.a. |
| --- | --- | --- | --- | --- | --- | --- | --- |
|  | …I have difficulty getting an overview |  |  |  |  |  |  |
|  | …I experience problems moving around in a familiar environment |  |  |  |  |  |  |
|  | …I have difficulty finding my way in a familiar environment |  |  |  |  |  |  |
|  | ...I experience problems moving about in an unfamiliar environment |  |  |  |  |  |  |
|  | …I have difficulty finding my way in an unfamiliar environment |  |  |  |  |  |  |
|  | …I have difficulty noticing traffic at intersections |  |  |  |  |  |  |
|  | …I find it hard to use public transportation |  |  |  |  |  |  |
|  | …I experience problems stepping up and down the sidewalk |  |  |  |  |  |  |
|  | …I find it hard to walk across uneven travel surfaces |  |  |  |  |  |  |
|  | …I have difficulty not to fall or stumble |  |  |  |  |  |  |
|  | …I have difficulty going out to for instance sporting events, cinema, friends, church, restaurants, places of entertainment, etc. |  |  |  |  |  |  |
|  | …I go out less often than I would want because of my vision at night |  |  |  |  |  |  |

Part 6

***The next items consider indoor situations***

|  | When there is poor or insufficient lighting... | never | sometimes | regularly | often | always | n.a. |
| --- | --- | --- | --- | --- | --- | --- | --- |
|  | …I have difficulty noticing the furniture in a room |  |  |  |  |  |  |
|  | …I have difficulty finding small objects |  |  |  |  |  |  |
|  | …I have difficulty reading the paper or a book |  |  |  |  |  |  |
|  | …in restaurants, I have difficulty reading the menu |  |  |  |  |  |  |
|  | …I have difficulty avoiding bumping into obstacles or persons |  |  |  |  |  |  |
|  | …I have difficulty recognizing faces |  |  |  |  |  |  |

Part 7

***The next items consider situations in general***

|  |  | never | sometimes | regularly | often | always | n.a. |
| --- | --- | --- | --- | --- | --- | --- | --- |
|  | I experience problems going from light to dark |  |  |  |  |  |  |
|  | If it is dark, I experience problems moving about in crowded situations |  |  |  |  |  |  |
|  | I feel blind at night |  |  |  |  |  |  |
|  | At a social event when there is dim light, I feel insecure |  |  |  |  |  |  |
|  | I feel restricted because of my vision at night |  |  |  |  |  |  |
|  | When it is dark, I feel dependent on others to help me because of my vision at night |  |  |  |  |  |  |
